# Supplementary material for: Nitrofuran Derivatives Cross-Resistance Evidence—Uropathogenic Escherichia coli Nitrofurantoin and Furazidin In Vitro Susceptibility Testing
Source: J Clin Med. 2023 Aug 8;12(16):5166. doi: 10.3390/jcm12165166 (PMC10455607; doi:10.3390/jcm12165166)
Supplement: Supplementary file 1 [file jcm-12-05166-s001.zip › jcm-2522354-supplementary.pdf]

Table S1. The data presented in the study – the susceptibility testing results for nitrofurantoin and furazidin using broth microdilution and disk diffusion methods.

Sample No.	Species name	Minimal inhibitory concentration [mg/L]		Growth inhibition zone diameter [mm]	
		NIT	FUR	NIT	FUR
1	*Escherichia coli*	16	4	17	16
2	*Escherichia coli*	8	8	21	18
3	*Escherichia coli*	32	8	19	12
4	*Escherichia coli*	4	4	21	15
5	*Escherichia coli*	16	0,5	23	22
6	*Escherichia coli*	32	8	20	13
7	*Escherichia coli*	16	16	19	15
8	*Escherichia coli*	16	8	18	16
9	*Escherichia coli*	8	2	22	19
10	*Escherichia coli*	16	4	18	16
11	*Escherichia coli*	32	16	18	12
12	*Escherichia coli*	8	4	19	16
13	*Escherichia coli*	32	64	17	6
14	*Escherichia coli*	256	512	8	6
15	*Escherichia coli*	16	4	20	16
16	*Escherichia coli*	16	1	23	23
17	*Escherichia coli*	32	16	15	11
18	*Escherichia coli*	16	4	20	20
19	*Escherichia coli*	32	4	22	17
20	*Escherichia coli*	32	4	20	14
21	*Escherichia coli*	32	4	20	16
22	*Escherichia coli*	16	8	19	15
23	*Escherichia coli*	16	4	19	14
24	*Escherichia coli*	32	8	20	17
25	*Escherichia coli*	16	32	14	9
26	*Escherichia coli*	32	16	16	13
27	*Escherichia coli*	16	8	21	16
28	*Escherichia coli*	32	16	19	10
29	*Escherichia coli*	128	64	10	6
30	*Escherichia coli*	64	64	11	9
31	*Escherichia coli*	128	256	13	6

32	*Escherichia coli*	8	8	20	16
33	*Escherichia coli*	16	16	20	16
34	*Escherichia coli*	16	8	20	16
35	*Escherichia coli*	8	4	21	19
36	*Escherichia coli*	32	8	15	16
37	*Escherichia coli*	512	256	8	7
38	*Escherichia coli*	16	8	21	16
39	*Escherichia coli*	16	32	21	15
40	*Escherichia coli*	16	16	20	15
41	*Escherichia coli*	32	4	23	19
42	*Escherichia coli*	32	64	18	10
43	*Escherichia coli*	128	16	10	8
44	*Escherichia coli*	1	2	29	24
45	*Escherichia coli*	16	32	20	12
46	*Escherichia coli*	8	4	25	19
47	*Escherichia coli*	512	256	10	6
48	*Escherichia coli*	32	16	17	10
49	*Escherichia coli*	128	128	10	9
50	*Escherichia coli*	8	8	21	17
51	*Escherichia coli*	8	4	20	17
52	*Escherichia coli*	16	16	17	17
53	*Escherichia coli*	16	32	17	12
54	*Escherichia coli*	64	64	16	10
55	*Escherichia coli*	16	8	18	16
56	*Escherichia coli*	128	256	10	6
57	*Escherichia coli*	128	128	9	6
58	*Escherichia coli*	64	16	17	10
59	*Escherichia coli*	8	4	15	11
60	*Escherichia coli*	64	32	18	9
61	*Escherichia coli*	4	4	20	18
62	*Escherichia coli*	8	8	21	16
63	*Escherichia coli*	64	32	15	9
64	*Escherichia coli*	16	1	19	18
65	*Escherichia coli*	256	128	6	6
66	*Escherichia coli*	128	64	10	6
67	*Escherichia coli*	4	4	28	23
68	*Escherichia coli*	8	8	19	16
69	*Escherichia coli*	32	64	14	10

70	*Escherichia coli*	16	8	21	17
71	*Escherichia coli*	32	8	19	15
72	*Escherichia coli*	16	16	19	15
73	*Escherichia coli*	8	8	21	16
74	*Escherichia coli*	16	16	19	14
75	*Escherichia coli*	64	16	14	10
76	*Escherichia coli*	128	128	9	6
77	*Escherichia coli*	16	8	21	20
78	*Escherichia coli*	16	8	19	14
79	*Escherichia coli*	8	2	19	17
80	*Escherichia coli*	16	4	17	16
81	*Escherichia coli*	16	8	19	15
82	*Escherichia coli*	128	32	11	9
83	*Escherichia coli*	8	2	22	20
84	*Escherichia coli*	8	4	21	20
85	*Escherichia coli*	8	4	21	17
86	*Escherichia coli*	8	4	21	15
87	*Escherichia coli*	32	8	19	11
88	*Escherichia coli*	32	4	16	12
89	*Escherichia coli*	16	16	19	10
90	*Escherichia coli*	16	8	20	18
91	*Escherichia coli*	8	2	21	16
92	*Escherichia coli*	4	1	24	19
93	*Escherichia coli*	32	8	19	13
94	*Escherichia coli*	32	4	19	15
95	*Escherichia coli*	8	2	20	14
96	*Escherichia coli*	32	16	18	13
97	*Escherichia coli*	32	1	19	16
98	*Escherichia coli*	16	2	20	16
99	*Escherichia coli*	32	4	19	14
100	*Escherichia coli*	16	4	22	14

NIT – nitrofurantoin; FUR - furazidin
